# *Cryptosporidium felis* Infection, Spain

**DOI:** 10.3201/eid1209.060385

**Published:** 2006-09

**Authors:** María Teresa Llorente, Antonio Clavel, Marzo Varea, María Pilar Goñi, Juan Sahagún, Susana Olivera

**Affiliations:** * Universidad de Zaragoza, Zaragoza, Spain

**Keywords:** Cryptosporidium felis, immunocompetent, human, SSU rRNA, letter

**To the Editor:** Coccidian protozoans that belong to the genus *Cryptosporidium* frequently cause gastrointestinal infection in humans and animals and are distributed worldwide. *Cryptosporidium hominis* and the cattle genotype of *C. parvum* are responsible for most human infections. However, other species and genotypes of *Cryptosporidium*, such as *C. felis*, *C. muris*, *C. meleagridis*, *C. canis*, *C. parvum* pig genotype, and *C. parvum* cervine genotype, have also been detected in stool samples of immunosuppressed and immunocompetent patients (*1*). Since 1999, when Pieniazek et al. described 3 cases of *C. felis* infection in HIV-positive patients (*2*), several studies have confirmed that this species can infect humans. Recently, Muthsusamy et al. described *C. felis* infections in 5 HIV-positive persons in southern India (*3*). In this article, we describe our experience with an imported case of *C. felis* infection in Spain.

A pediatrician requested a parasitologic study for an immunocompetent, 4-year-old boy with diarrhea. The child came from an orphanage in Calcutta, India; he had arrived in Spain 10 days earlier after having been adopted by a Spanish family. Stool specimens were tested for a wide panel of enteric pathogens, including bacteria, viruses, and parasites. *Cryptosporidium* oocysts were detected by direct microscopic visualization of the samples, which had been concentrated by formalin–ethyl acetate sedimentation and stained with a modified Ziehl-Neelsen stain. Results were also positive for *Cryptosporidium* for samples tested by using an immunochromatographic (Crypto-Strip, Coris Bioconcept, Gembloux, Belgium) (*4*) and an immunofluorescent assay (Merifluor *Cryptosporidium*/*Giardia*, Meridian Diagnostics, Cincinnati, OH, USA).

DNA was extracted as described elsewhere (*5*), purified with polyvinyl-pyrrolidone, and stored at –20°C in Tris-EDTA buffer. After DNA extraction, PCR–restriction fragment length polymorphism (RFLP) analysis was performed by using previously described protocols based on the small subunit (SSU) rRNA gene (*6*), with digestion of the amplicon by the restriction enzymes *Ssp*I for species diagnosis or *Vsp*I for *C. parvum* genotype identification. For DNA sequencing, PCR products of the 18S rRNA gene fragments were purified and used for direct sequencing in an ABI377 automated sequencer (Applied Biosystems, Foster City, CA, USA).

RFLP analysis showed a profile distinct from those of *C. hominis* and *C. parvum* cattle genotype and consistent with the published patterns for *Cryptosporidium felis*: 426 and 390 bp with *Ssp*I digestion; 476, 182, and 104 bp with *Vsp*I (*6*). The sequence of the PCR product was determined, and a comparison with all SSU rDNA *Cryptosporidium* sequences available in databanks showed 100% similarity with the homologous fragment of *C. felis* (GenBank accession no. AF112575).

To date, >30 cases of human infection by *C. felis* have been reported in the literature. Only 3 of them have occurred in immunocompetent patients: 2 in the United Kingdom (*7*) and 1 in Peru (*8*). To our knowledge, this is the first case of human *C. felis* infection diagnosed in Spain. The child had been in Spain for only 10 days, no pet animals lived in his new home, and he had not gone to kindergarten. Consequently, the infection was likely acquired in India.

The transmission route for the unusual *Cryptosporidium* species is unclear. In the study by Matos et al., only 1 of 4 immunocompromised patients with *C. felis* had been in close contact with cats at home (*9*). Unusual cryptosporidial infections are not restricted to immunocompromised hosts, and further investigation of the pathogenicity and epidemiology of these infections is necessary to establish their effect on public health and to identify risk factors for exposure and measures for prevention. The identification of species other than *C. hominis* and *C. parvum* that infect humans, and the transmission routes of such agents, has relevance for better understanding of the epidemiologic features of cryptosporidiosis.
